# Differential Phosphorylation of the Glucocorticoid Receptor in Hippocampal Subregions Induced by Contextual Fear Conditioning Training

**DOI:** 10.3389/fnbeh.2020.00012

**Published:** 2020-02-13

**Authors:** Renata Ponce-Lina, Norma Serafín, Martha Carranza, Carlos Arámburo, Roberto A. Prado-Alcalá, Maricela Luna, Gina L. Quirarte

**Affiliations:** ^1^Departamento de Neurobiología Conductual y Cognitiva, Instituto de Neurobiología, Universidad Nacional Autónoma de México, Querétaro, Mexico; ^2^Departamento de Neurobiología Celular y Molecular, Instituto de Neurobiología, Universidad Nacional Autónoma de México, Querétaro, Mexico

**Keywords:** memory, glucocorticoids, corticosterone, serine 232, serine 246

## Abstract

Aversive events induce the release of glucocorticoid stress hormones that facilitate long-term memory consolidation, an effect that depends on the activation of glucocorticoid receptors (GRs). GRs are distributed widely in the hippocampus. The dorsal region of the hippocampus has been related to cognitive functions and the ventral region to stress and emotion. GR acts as a transcription factor which after hormone binding becomes phosphorylated, affecting its cellular distribution and transcriptional activity. Two functionally well-described GR phosphorylation sites are serine 232 (pSer232), which enhances gene expression, and serine 246 (pSer246), having the opposite effect. Since gene expression is one of the plastic mechanisms needed for memory consolidation, we investigated if an aversive learning task would induce GR phosphorylation in the dorsal (DH) and the ventral (VH) hippocampus. We trained rats in contextual fear conditioning (CFC) using different foot-shock intensities (0.0, 0.5, or 1.5 mA). One subgroup of animals trained with each intensity was sacrificed 15 min after training and blood was collected to quantify corticosterone (CORT) levels in serum. Another subgroup was sacrificed 1 h after training and brains were collected to evaluate the immunoreactivity (IR) to GR, pSer232 and pSer246 by SDS-PAGE/Western blot in DH and VH, and by immunohistochemistry in dorsal and ventral CA1, CA2, CA3, and dentate gyrus (DG) hippocampal regions. The conditioned freezing response increased in animals trained with 0.5 and 1.5 mA during training and extinction sessions. The degree of retention and CORT levels were directly related to the intensity of the foot-shock. Although total GR-IR remained unaffected after conditioning, we observed a significant increase of pSer246-IR in the dorsal region of CA1 and in both dorsal and ventral DG. The only region in which pSer232-IR was significantly elevated was ventral CA3. Our results indicate that fear conditioning training is related to GR phosphorylation in specific subregions of the hippocampus, suggesting that its transcriptional activity for gene expression is favored in ventral CA3, whereas its repressor activity for gene-silencing is increased in dorsal CA1 and in both dorsal and ventral DG.

## Introduction

Organisms associate aversive stimuli with different cues present in the environment as part of adaptation mechanisms (Korte, 2001; Steimer, 2002). The molecular processes involved in this type of learning and subsequent memory formation are commonly studied with the use of different learning tasks such as contextual fear conditioning (CFC), in which a neutral stimulus (conditioned stimulus, CS), in this case a particular context, is associated with an aversive stimulus (unconditioned stimulus, US), usually a foot-shock (Jacobs et al., 2010).

It is known that the hippocampus is involved in the neural circuit of CFC. Several reports have established a functional differentiation along the dorsoventral axis of the rat hippocampus during fear conditioning, in such a way that the dorsal region is involved in the association of the contextual CS with the US (Phillips and LeDoux, 1992; Maren, 2001), whereas the ventral region is selective for the association of discrete cues with the US, and it has direct connections with the amygdala, which is also related to stress and anxiety (Maren and Holt, 2004; Trivedi and Coover, 2004; Yoon and Otto, 2007; Jacobs et al., 2010).

An increase of blood corticosterone (CORT) hormone levels is seen after CFC as a component of the stress response triggered by the aversive US. This increase, which is dependent on the foot-shock intensity (Cordero et al., 1998), leads to CORT binding to mineralocorticoid (MR) and glucocorticoid (GR) receptors. Although both receptors are involved in processing of fear memories (Cordero and Sandi, 1998; Donley et al., 2005; Brinks et al., 2009; Zhou et al., 2011), MRs are supposed to be saturated at this point due to their higher affinity to the hormone than that of GRs (Sandi, 2003); since we are interested in the study of the effects of CORT levels on memory after a stressful experience, the present study focuses on the activation of GRs.

Blocking the activation of GRs (by administration of antagonists or by genetic mutation of the GR) in several brain areas, including the amygdala, the hippocampus, and the prefrontal cortex impairs memory consolidation of CFC (Pugh et al., 1997; Donley et al., 2005; Revest et al., 2005; Rodrigues and Sapolsky, 2009), and of other learning tasks with an aversive component, such as water maze and inhibitory avoidance (Roozendaal and McGaugh, 1997). It is known that the dorsal hippocampus (DH) is involved in spatial information processing, and that the administration of a GR antagonist before or immediately after training into the ventral hippocampus (VH) impairs contextual fear memory consolidation (Donley et al., 2005). Considering that GR distribution is heterogeneous throughout CA1, CA2, CA3 and the dentate gyrus (DG) regions (Sarabdjitsingh et al., 2010), it might be possible that the requirement of GR activation for memory consolidation is not only different in DH from VH, but also among the different hippocampal subregions.

The GR is a transcription factor that, upon hormone binding, can be phosphorylated at several serine residues which are highly conserved in humans and rodents (Blind and Garabedian, 2008; Kadmiel and Cidlowski, 2013). In rats, a higher amount of phosphorylated serine 232 (pSer232) induces activation and nuclear translocation of the receptor and increases its transcriptional activity. By contrast, the phosphorylation of the serine 246 (pSer246) induces the opposite effect by promoting nuclear exportation of the GR or by its binding to co-repressors inside the nucleus, thus repressing transcription (Rogatsky et al., 1998; Wang et al., 2002; Adzic et al., 2009).

Because gene expression is involved in memory plasticity (Martin et al., 2000; Lamprecht and LeDoux, 2004; Sutton and Schuman, 2006), it is likely that the phosphorylation status of GR is altered during the acquisition process of CFC, affecting, in turn, its transcriptional activity. In this study we used semi-quantitative techniques to evaluate the amount of total GR protein, as well as the relative proportion of pSer232, and pSer246 variants in different subregions of the DH and VH of rats trained in CFC. Infusions of sodium channel blockers (Quiroz et al., 2003) and of transcription and translation inhibitors (Medina et al., 2019) into DH produce a strong amnestic effect when moderate foot-shock intensities are used for training of inhibitory avoidance, while no such effect is observed when a relatively strong foot-shock is used, suggesting that the involvement of the hippocampus in aversive memory is dependent upon the intensity of the learning experience. In order to know if GR phosphorylation depends upon the strength of training, we also evaluated the phosphorylated status of the GR in rats trained with low and high foot-shock intensity. This information will help us understand the molecular processes whereby glucocorticoids (GCs) exert their effects on fear memory consolidation.

## Materials and Methods

### Subjects

Male adult Wistar rats (*Rattus norvegicus albinus*), obtained from the breeding colony of the Instituto de Neurobiología, Universidad Nacional Autónoma de México, were carried to the vivarium of our laboratory and placed in individual acrylic home-cages (24 × 21 × 45 cm), with *ad libitum* water and food, with a light/dark cycle of 12/12 h (lights on at 7:00 am) and constant temperature of 23 ± 1°C in the room. These experimentally naïve animals were maintained undisturbed for 3 days to allow them to adapt to the new housing conditions. Animals weighed between 250 and 350 g at the beginning of the experiments. All animals were treated in accordance with the (NORMA Oficial Mexicana NOM-062-ZOO-1999, 2001), following the specifications for the production, care and use of animals in the laboratory, as well as the recommendations of the Guide for Care and Use of Laboratory Animals of the National Research Council (National Research Council, 2011). The protocols for these experiments were approved by the Ethics Committee of the Instituto de Neurobiología, Universidad Nacional Autónoma de México.

### Habituation, Contextual Fear Conditioning, and Extinction

#### Apparatus

The CFC chamber (H10-11R-TC, Coulbourn Instruments, Whitehall, PA, USA, 30.48 × 25.4 × 30.48 cm) has transparent acrylic back and front walls, and steel side panels. The grid floor has electrifiable stainless steel-bars (0.5 cm in diameter, separated by 1.0 cm) connected to a shock generator (H13-15, Coulbourn). A digital camera (SenTech, Carrollton, TX, USA) was located on the ceiling of the chamber, and a red light and a white light were located on the opposite sidewalls. Both the shock generator and the lights were connected to a USB interface (ACT-710, Actimetrics, Wilmette, IL, USA), which, along with the camera, were controlled by the FreezeFrame (Actimetrics) software installed in a computer running Microsoft Windows XP. To avoid auditory disturbances, a speaker emitting white noise (60 dB) was present during all behavioral procedures. The chamber was located inside a sound-attenuating cubicle (Med Associates Inc., USA) located in a sound-attenuated room.

#### Behavioral Procedures

All the behavioral procedures were performed between 0800 h and 1400 h to avoid the peak of glucocorticoid release in the experimental subjects. One hour before each session, rats were placed in a rack near the room in which conditioning took place, and at the end of the sessions they were returned to the rack and remained there for 1 h, and then were carried back to the vivarium. Each animal was handled for 5 min during three consecutive days. On the next day, on the habituation session, each rat was allowed to explore the chamber for 20 min, with all the apparatuses turned on. Twenty-four hours after habituation, during the training session, the rats could explore the chamber for 3 min (pre-shock), and immediately after the third minute, one foot-shock (1 s) per minute was delivered eight times.

Rats were randomly assigned to one of three independent groups that received 0.0 (*n* = 29), 0.5 (*n* = 31), or 1.5 (*n* = 31) mA foot-shocks. One minute after the last foot-shock, animals were returned to their home cages. To evaluate if memory strength was related to the foot-shock intensity, one subgroup of animals that had been trained with each foot-shock intensity (*n* = 11 per subgroup) was returned to the same chamber 48 h post-training and remained there for 11 min without foot-shock (extinction). The remaining animals were sacrificed after training for biochemical procedures, as described below. The interior of the chamber was cleaned with 10% ethanol after each subject had occupied it.

All sessions were recorded and analyzed for freezing behavior, defined as the absence of movements except for those required for breathing (Maren and Fanselow, 1997), using the FreezeFrame software (Actimetrics). The bout length (change in pixels/frame) was set to 0.75 s and the threshold for freezing behavior was determined individually for each rat. During training, the freezing response was measured 10 s after each foot-shock to avoid the hyperactivity that is produced immediately after the administration of the foot-shocks.

### Biochemical Procedures

As stated above, each of the 0.0 mA, 0.5 mA, and 1.5 mA trained groups was divided into four subgroups that were tested for extinction (*n* = 11 per group), or for CORT measurement (*n* = 8 per group), Western blotting (*n* = 4, 5, and 5 per group, respectively), or for immunohistochemistry (*n* = 6, 7, and 7 per group, respectively); these rats were sacrificed by decapitation, and their brains were collected. Three additional control groups of non-stressed animals that were handled but not shocked (Handled groups) were also used for CORT measurement (*n* = 7), Western blotting (*n* = 5), and immunohistochemistry (*n* = 7); these Handled groups were sacrificed 2 days after the last session of handling, on the same day and time that the trained rats were sacrificed.

### Corticosterone Measurement

For CORT measurement the trained rats were sacrificed 15 min after training and trunk blood was collected. Blood samples were centrifuged at 2,000 rpm for 30 min at 4°C and were collected and stored at −80°C until they were analyzed. Serum CORT levels were measured using a commercial kit (Corticosterone ELISA kit, Enzo Life Sciences, Farmingdale, NY, USA). The provided steroid displacement reagent was used to dilute the serum samples, such that 2.5 parts of steroid displacement reagent were present for every 97.5 parts of the undiluted sample. The remainder of the protocol was conducted in accordance with the manufacturer’s instructions. Absorbance at 405 nm was read using a microplate reader (iMarK, Bio-Rad, Hercules, CA, USA).

### Semi-quantification of Total GR and Phosphorylated GR Variants

#### Western Blotting

For Western blotting the trained rats were sacrificed 1 h after training and their brains were immediately frozen in cold isopentane and stored at −80°C. DH and VH were punched out from both hemispheres with fine-tip scissors at –21°C in a cryostat (Leica), and the samples were individually stored in separate tubes. Each sample was homogenized with a sonicator (Cole Palmer) in 60 μl RIPA lysis buffer (Cell Signaling Technology, Inc., Danvers, MA, USA) containing a COMPLETE protease inhibitor cocktail (Cell Signaling Technology, Inc., Danvers, MA, USA) and shaken for 2 h at 4°C. Homogenates were centrifuged at 10,000 *g* for 15 min at 4°C and supernatants were collected. Protein concentration was determined by Bradford assay (Bradford, 1976), and 50 μg of total protein of each sample was resolved by SDS-PAGE electrophoresis in 10% polyacrylamide gels under reducing conditions at 100 V (Laemmli, 1970), and then transferred for 1 h at 200 mA to membranes of nitrocellulose (Bio-Rad, Hercules, CA, USA). Membranes were blocked in 5% nonfat dry milk (Bio-Rad, Hercules, CA, USA) in Tris-buffered saline (TBS) during 2 h, and then incubated overnight at room temperature with either a rabbit anti-GR phospho-S211 antibody (1:1,000; Cell Signaling Technology, Inc., Danvers, MA, USA, Cat. #4161) that recognizes rat pSer232, or a rabbit anti-GR phospho-S226 antibody (1:800; Abcam Inc., Cambridge, MA, USA, Cat. #ab93104) that recognizes rat pSer246. Blots were incubated with a goat anti-rabbit secondary antibody conjugated to horseradish peroxidase (1:4,000; Invitrogen) for 2 h at room temperature. Immunoreactive bands were developed by chemiluminescence using ECL (Amersham Biosciences, Montreal) on hyper film (Amersham; Buckinghamshire, UK). For determination of total GR and loading control (α-tubulin), membranes were stripped in a buffer containing 62.5 mM TRIS-HCl, pH 6.7, SDS 2% and 100 mM β-mercaptoethanol, at 65°C for 30 min, washed several times with Tween-TBS (TTBS), reblocked for 2 h, and incubated overnight at room temperature with either an anti-GR-H300 antibody (1:2,000; Santa Cruz Biotechnology Inc., Santa Cruz, CA, USA, Cat. #sc-8992) or an anti-α-tubulin antibody (1:5,000; Abcam, Cat. #ab24246). Secondary antibody and chemiluminescence development protocols were similar to those described above. Immunoreactive protein bands were quantified by densitometric analysis using Image Lab (Bio-Rad, Hercules, CA, USA) software.

#### Immunohistochemistry

For the immunohistochemical procedure the rats were sacrificed by decapitation 1 h after training, and their brains were collected. Brains were immediately frozen in cold isopentane and stored at −80°C. Brains were sectioned coronally at 20 μm thickness on a cryostat (Leica). Sections containing DH and VH were mounted on Superfrost slides (Thermo Fisher) and immediately fixed with 4% fresh paraformaldehyde for 10 min. Epitope exposure took place in citrate buffer (10 mM sodium citrate, Triton 0.05%, pH 6) at 80°C for 30 min. The sections were blocked in 5% nonfat dry milk for 2 h and serial sections were double-labeled for total GR and for either of the two phosphorylated GR variants, using a mouse anti-GR monoclonal antibody (1:1,500; Abcam, Cat. #ab2768) and either a rabbit anti-pSer211-GR antibody (1:200; Cell Signaling Technology, Inc., Danvers, MA, USA, Cat. #4161) that recognizes rat pSer232, or a rabbit anti-pSer226-GR antibody (1:400; Abcam, Cat. #ab93104) that recognizes rat pSer246, incubated overnight at 4°C in a humid chamber. To reduce background noise, slides were incubated with 1% Sudan Black (Sigma) for 30 min. Secondary antibodies employed were as follows: rabbit anti-mouse conjugated with FITC (1:200; Invitrogen, Cat. #31561) and goat anti-rabbit conjugated with Cy3 (1:5,000; Invitrogen, Cat. #A10520). Nuclei were stained with 4′,6-diamidino-2-phenylindole (DAPI; 500 ng/mL; Invitrogen). Slides were covered with mounting medium VectaShield (Vector Laboratories, Burlingame, CA, USA). Images were acquired with a confocal microscope LSM 510 (Carl Zeiss) at 50× magnification in one focal plane, with lasers at excitation wavelengths of 488 nm (for FITC), 561 nm (for Cy3), and a Coherent-XR multiphotonic laser at 350 nm (for DAPI). Three frames were acquired for CA1, CA2, CA3, and the DG for both DH and VH, using the same microscope parameters. The data were analyzed with ImageJ (NIH, USA) software.

### Statistical Analyses

Behavioral data were presented as the minute by minute percentage of freezing (mean ± SEM) in each session and was analyzed with a two-way ANOVA, with time as Factor A and foot-shock intensity as Factor B. CORT serum levels and optical densities (Western blot densitometry) were analyzed with a one-way ANOVA. Percentage of immunoreactive cells was analyzed with a two-way ANOVA, with the hippocampal area as Factor A and the foot-shock intensity as Factor B. When appropriate, the Fisher LSD was used as a *post hoc* test for pairwise comparisons. A *P*-value of less than 0.05 was considered statistically significant. The data were analyzed with SigmaPlot (Systat Software, San Jose, CA, USA).

## Results

### Contextual Fear Conditioning

**Figure 1:**
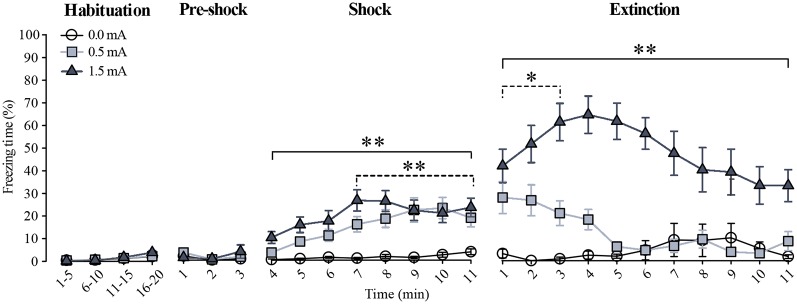
Freezing behavior during habituation, training, and extinction. Data are expressed as the percentage of freezing time (mean ± SEM). For the habituation session, the freezing response was grouped in 5 min blocks. For training (pre-shock and shock) and extinction sessions, the percentage of freezing is shown minute by minute. Dotted lines indicate the minutes during which the 0.5 mA group was different from the 0.0 mA group, whereas the continuous lines indicate the minutes during which the 1.5 mA group was different from the 0.0 mA group. All rats studied in this report (animals sacrificed after training and animals that were tested through extinction) are included in the habituation and the training sessions in this graph: 0.0 mA (*n* = 29), 0.5 mA (*n* = 31), and 1.5 mA (*n* = 31). Some animals from these groups were further tested through the extinction session: 0.0 mA (*n* = 11), 0.5 mA (*n* = 11) and 1.5 mA (*n* = 11; see text for details). *p < 0.01, **p < 0.001 vs. the 0.0 mA group.

The percentage of freezing time in rats exposed to CFC is shown in Figure 1. No freezing behavior was seen during the habituation session in any of the groups (*F*_(2,1760)_ = 2.895, *p* = 0.056); on the second session, lack of freezing was also observed during the first 3 min where the animals were re-exposed to the context before foot-shock administration (*F*_(2,264)_ = 1.237, *p* = 0.292), indicating that the context inside the chamber was not stressful. When the foot-shocks were administered significant foot-shock (*F*_(2,704)_ = 86.076, *p* < 0.001) and time (*F*_(7,704)_ = 6.242, *p* < 0.001) effects became evident, as well as a significant foot-shock × time interaction (*F*_(14,704)_ = 1.907, *p* < 0.05). The Fisher LSD showed that, when compared to the 0.0 mA group, the freezing response of the 1.5 mA group was evident right after the first foot-shock (*p* < 0.001 vs. 0.0 mA), whereas the 0.5 mA group started freezing significantly after the seventh foot-shock (*p* < 0.001 vs. 0.0 mA). The 1.5 and 0.5 mA groups differed from each other on minutes 6 and 7 (*p* < 0.001), where the animals trained with the 1.5 mA intensity showed a higher freezing response.

To find out whether the higher foot-shock intensity produced a stronger memory of the task, we investigated resistance to extinction of freezing on the third 11-min session, run 48 h after training, as resistance to extinction is an objective measure of the strength of learning. During this session there were significant time (*F*_(10,330)_ = 2.043, *p* < 0.05) and foot-shock (*F*_(2,330)_ = 169.441, *p* < 0.001) effects, as well as a significant time × foot-shock interaction (*F*_(20,330)_ = 2.262, *p* < 0.01). The Fisher LSD showed that the 1.5 mA group had a higher freezing response than the 0.0 mA and the 0.5 mA groups at the first (*p* < 0.001) and second (*p* < 0.001) minutes, respectively; these differences remained throughout the rest of the session; the 0.5 mA group only differed from the 0.0 mA group during the first 3 min of this session (*p* < 0.01). These results showed that animals trained with 0.5 and 1.5 mA learned the task, and that memory retention of the CFC was stronger and lasted longer in the group trained with the higher foot-shock intensity.

### Corticosterone Quantification

**Figure 2:**
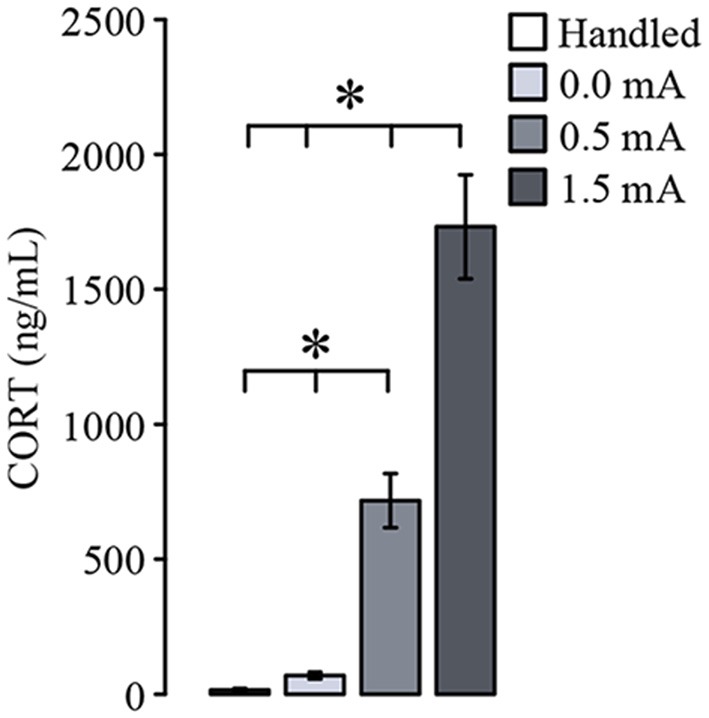
Mean (± SEM) of corticosterone (CORT) serum concentration measured 15 min after training, and 2 days after the last handling session for the Handled group (*n* = 7). Animals were trained with 0.0 mA (*n* = 8), 0.5 mA (*n* = 8) or 1.5 mA (*n* = 8). Serum CORT concentration is related to the intensity of the foot-shock used for contextual fear conditioning. *p < 0.001.

To verify that CORT levels in serum are increased after CFC (Cordero et al., 1998), we quantified the concentration of this hormone 15 min after training. The ANOVA showed a significant difference among the groups (*F*_(3,27)_ = 50.819, *p* ≤ 0.001). As depicted in Figure 2, both the group trained with 0.0 mA and the Handled group showed very low serum CORT concentrations, indicating that the context inside the chamber was not a stressor. As we had expected, the groups trained with 0.5 and 1.5 mA showed a higher amount of CORT than the Handled (*p* < 0.001 for each comparison) and the 0.0 mA (*p* < 0.001 for each comparison) groups, and we also found that CORT levels in serum were positively related to the intensity of the foot-shock, because there was a statistical difference between the 0.5 and 1.5 mA groups (*p* < 0.001). These results show that the training session led to a significant increase of CORT in serum that was related to the intensity of training.

### Proportion of Total and Phosphorylated Variants of GR in Hippocampal Regions

**Figure 3:**
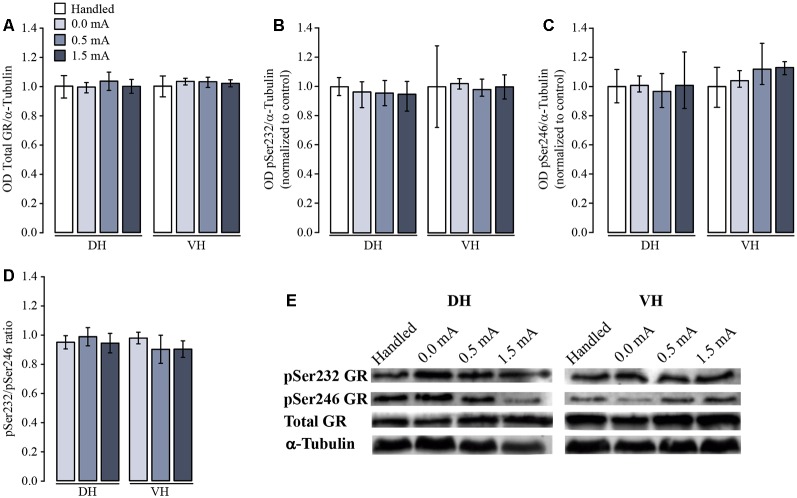
Western blot and densitometric analysis of total and phosphorylated variants of glucocorticoid receptor (GR). Rats were sacrificed 1 h after training with 0.0 mA (*n* = 4), 0.5 mA (*n* = 5) or 1.5 mA (*n* = 5), and 2 days after the last handling session for the Handled group (*n* = 5). Data are expressed as the mean (±SEM) of **(A)** optical density (OD) of total GR; **(B)** proportion of pSer232; **(C)** proportion of pSer246; **(D)** pSer232/pSer246 ratio in dorsal (DH) and ventral (VH) hippocampus; these data were obtained dividing the OD of each immunoreactive band by the OD of the loading control α-tubulin, and normalized to the OD of the Handled group. **(E)** Representative blots for each signal in DH and VH.

As a first approach to studying a possible effect of CFC training upon the total amount and the relative proportion and distribution of GR, pSer232, and pSer246, we performed a densitometric analysis by Western blotting of DH and VH samples. Total GR immunoreactivity (IR) did not differ among groups in either DH (*F*_(3,20)_ = 0.468, *p* = 0.708) or VH (*F*_(3,20)_ = 1.52, *p* = 0.240; Figures 3A,E). The proportion of pSer232-IR was not altered in DH (*F*_(3,20)_ = 0.357, *p* = 0.785) nor in VH (*F*_(3,20)_ = 0.481, *p* = 0.699) after training (Figures 3B,E). Likewise, the proportion of pSer246-IR did not change after training in either DH (*F*_(3,12)_ = 0.091, *p* = 0.963) or VH (*F*_(3,8)_ = 1.609, *p* = 0.262; Figures 3C,E).

Some reports indicate that the resulting transcriptional activity of the GR depends on the proportion between the two phosphorylated variants. For this reason, we also calculated the ratio between pSer232-IR and pSer246-IR for all treatments and we did not find a significant effect in either the DH (*F*_(2,9)_ = 0.166, *p* = 0.849) or the VH (*F*_(2,6)_ = 0.414, *p* = 0.679; Figure 3D).

### Cellular Distribution of Total and Phosphorylated Variants of GR in Dorsal and Ventral Hippocampal Subregions

**Figure 4:**
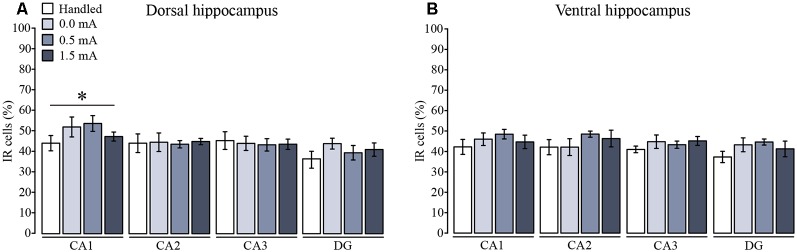
Mean (±SEM) percentage of immunoreactive cells for total GR in the subregions of **(A)** the dorsal and **(B)** the ventral hippocampus. Rats were sacrificed 1 h after training with 0.0 mA (*n* = 6), 0.5 mA (*n* = 7), or 1.5 mA (*n* = 7), and 2 days after the last handling session for the Handled group (*n* = 7). Data were obtained by averaging the percentage of immunoreactive (IR) cells to total GR relative to the total number of nuclei stained with DAPI in three different fields of CA1, CA2, CA3, and dentate gyrus (DG). There was a significant subregion effect where CA1 had a higher percentage of IR cells than the rest of the subregions (*p < 0.05 vs. each of the other subregions).

In order to make a more localized analysis of the cellular distribution of Total GR, pSer232, and pSer246 IR throughout the DH and VH regions, we measured the specific distribution of total GR and phosphorylated GR variants in the different subregions of DH and VH, and a semi-quantitative analysis was made by counting the number of immunoreactive cells for each specific antibody in CA1, CA2, CA3, and DG. We found a significant subregion effect on the number of immunoreactive cells for total GR among the DH subregions (*F*_(3,96)_ = 4.53, *p* = 0.005). Fisher LSD showed that CA1 had more immunoreactive cells for total GR than CA2, CA3 and DG (*p* < 0.05 for each comparison; Figure 4A); but there was no significant effect of the foot-shock (*F*_(3,96)_ = 0.748, *p* = 0.526), nor a significant interaction between the percentage of immunoreactive cells and the treatments (*F*_(9,96)_ = 0.558, *p* = 0.828). Moreover, this approach confirmed that there was no change in total GR-IR in any of the VH subregions (*F*_(3,92)_ = 1.34, *p* = 0.266) and also the effect of the foot-shock intensity was not significant (*F*_(3,92)_ = 2.470, *p* = 0.067), so there was no interaction among ventral subregions and shock intensity (*F*_(9,92)_ = 0.330, *p* = 0.963; Figure 4B).

**Figure 5:**
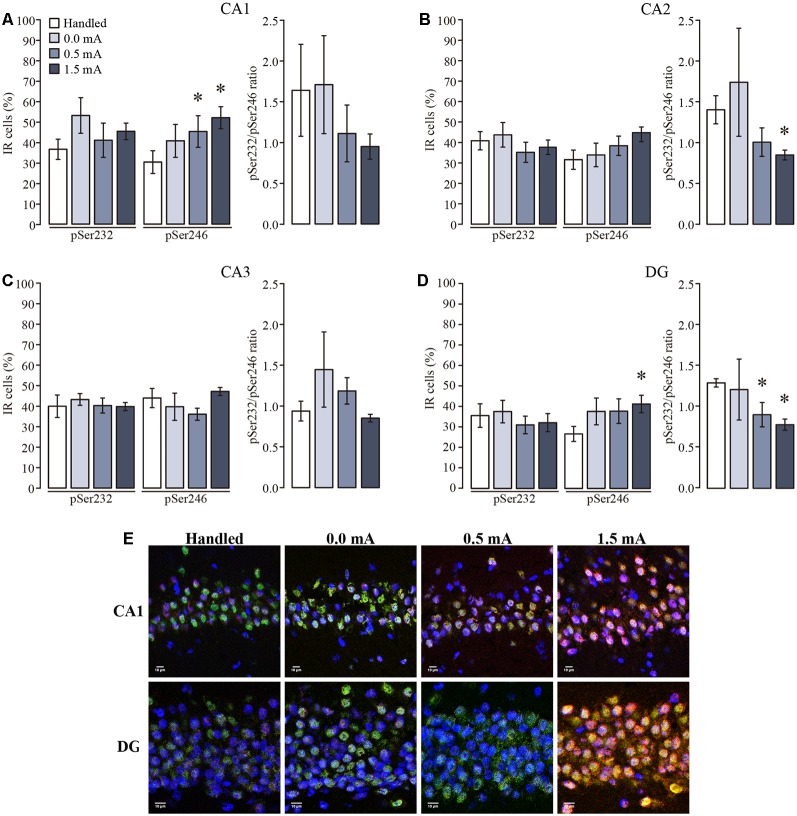
Mean (±SEM) percentage of immunoreactive cells for pSer232 and pSer246 in the subregions of the dorsal hippocampus. Rats were sacrificed 1 h after training with 0.0 mA (*n* = 6), 0.5 mA (*n* = 7) or 1.5 mA (*n* = 7), and 2 days after the last handling session for the Handled group (*n* = 7). Data were obtained by averaging the percentage of immunoreactive (IR) cells to pSer232 or pSer246, relative to the total number of nuclei stained with DAPI in **(A)** CA1; **(B)** CA2; **(C)** CA3; and **(D)** and DG. The pSer232/pSer246 ratio is shown on the right side of each plot. **(E)** Representative micrographs of CA1 and DG, with the signal of total GR seen in green, pSer246 in red, nuclei in blue, and co-localization of total GR and pSer246 signals are visualized in yellow. *p < 0.05 vs. Handled.

In the DH all groups showed that immunoreactive cells for pSer232 were not different among subregions (*F*_(3,92)_ = 2.403, *p* = 0.073), and were not affected by the treatments (*F*_(3,92)_ = 1.042, *p* = 0.378), and the interaction between those factors was not significant (*F*_(9,92)_ = 0.333, *p* = 0.962; Figures 5A–D). Likewise, the percentage of immunoreactive cells for pSer246 did not differ among subregions (*F*_(3,92)_ = 1.428, *p* = 0.240), but it was affected by treatments (*F*_(3,92)_ = 4.429, *p* = 0.006), although the interaction between both factors was not significant (*F*_(9,92)_ = 0.767, *p* = 0.647). Fisher LSD showed that 0.5 and 1.5 mA groups had more immunoreactive cells for pSer246 in CA1 as compared with the Handled group (*p* < 0.05; Figure 5A), while no differences were found in the percentage of immunoreactive cells for pSer246 in CA2 nor in CA3 among treatments (Figures 5B,C). Although the ratio between the IR of both phosphorylated GR variants also decreased in the group trained with 1.5 mA in CA2 (*p* < 0.05), we did not observe a significant increase of immunoreactive cells for pSer246 in this group; instead we observed a tendency to increase in comparison to the Handled group (Figure 5B). In DG there were more immunoreactive cells for pSer246 in the 1.5 mA group than in the Handled group (*p* < 0.05; Figure 5D). Interestingly, we found an effect of training on the ratio of pSer232 relative to pSer246 (*F*_(3,92)_ = 3.488, *p* = 0.019); multiple comparisons between groups showed that this ratio decreased after training with 0.5 and 1.5 mA as compared with the Handled group (*p* < 0.05 for each comparison). As shown in the micrograph (Figure 5E), it was an increase in the IR to pSer246 in CA1 and in DG after training, as well as a greater colocalization with total GR-IR inside the cell nuclei.

**Figure 6:**
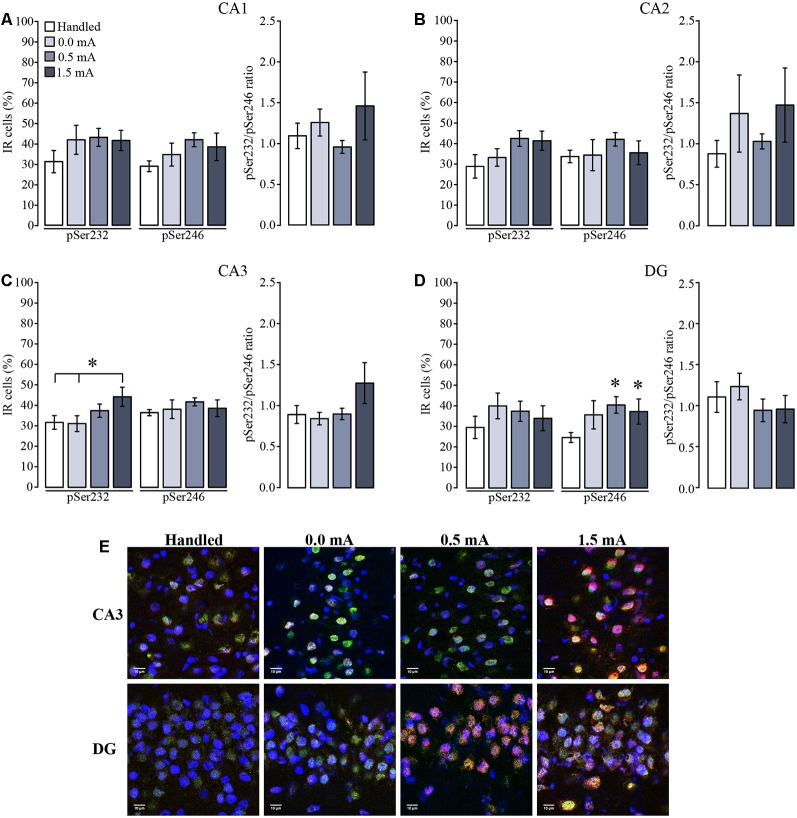
Mean (±SEM) percentage of immunoreactive cells for pSer232 and pSer246 in the subregions of the ventral hippocampus. Rats were sacrificed 1 h after training with 0.0 mA (*n* = 6), 0.5 mA (*n* = 7) or 1.5 mA (*n* = 7), and 2 days after the last handling session for the Handled group (*n* = 7). Data were obtained by averaging the percentage of immunoreactive (IR) cells to pSer232 or pSer246, relative to the total number of nuclei stained with DAPI in **(A)** CA1; **(B)** CA2; **(C)** CA3; and **(D)** DG. The pSer232/pSer246 ratio is shown on the right side of each plot. **(E)** Representative micrographs of CA3 and DG, with the signal of total GR seen in green, pSer232 (in CA3) and pSer246 (in DG) in red, nuclei are seen in blue, and co-localization of total GR with pSer232 (in CA3) and pSer246 (in DG) signals is visualized in yellow. *p < 0.05 vs. Handled.

In the VH, percentage of immunoreactive cells for pSer232 of each group did not change among subregions (*F*_(3,92)_ = 0.523, *p* = 0.667), but were affected due to the treatments (*F*_(3,92)_ = 3.657, *p* = 0.015), although the interaction between both factors was not significant (*F*_(9,92)_ = 0.580, *p* = 0.810; Figures 6A–D). *Post hoc* analysis showed no differences in immunoreactive cells for pSer232 in CA1 nor in CA2 among treatments (Figures 6A,B). This analysis also showed an increase in immunoreactive cells for pSer232 in CA3 in the 1.5 mA trained group when compared with the Handled and the 0.0 mA groups (*p* < 0.05 for each comparison; Figure 6C). Similarly, the cellular distribution of pSer246-IR of each group did not change among subregions (*F*_(3,92)_ = 0.603, *p* = 0.615 ), but it was affected by the treatment (*F*_(3,92)_ = 3.842, *p* = 0.012), although there was no significant interaction between both factors (*F*_(9,92)_ = 0.336, *p* = 0.961). Multiple comparisons among groups showed that the 0.5 and 1.5 mA groups had higher percentage of immunoreactive cells for pSer246 than the Handled group in the DG (*p* < 0.05 for each comparison; Figure 6D). The ratio between pSer232 and pSer246 IR of each group did not change among subregions (*F*_(3,91)_ = 0.730, *p* = 0.537, nor due to treatments (*F*_(3,91)_ = 1.831, *p* = 0.147). As shown in the micrograph (Figure 6E), there was an increase in the IR to pSer232 and pSer246 in CA3 and in DG after training, respectively, as well as a greater co-localization with total GR-IR inside the nuclei of cells.

## Discussion

The protocol used for CFC in this study demonstrated that the degree of retention of the task was directly related to the intensity of the foot-shock, as described elsewhere (Fanselow and Bolles, 1979; Cordero et al., 1998; Luyten et al., 2011), inducing freezing percentages very similar to those reported in the literature with the same shock intensities (Phillips and LeDoux, 1992; Kim et al., 1993; Levenson et al., 2002; Wiltgen et al., 2006; Suvrathan et al., 2014).

In addition to causing different levels of freezing during retention, the two shock intensities used for CFC training also stimulated CORT release into the bloodstream that was proportional to the intensity of the shock. This effect had been observed previously with this task (Cordero et al., 1998), as well as in other learning paradigms whose protocols used different intensities of aversive stimulation for training (Armario et al., 1986; Pitman et al., 1988; Sandi et al., 1997; Rodgers et al., 1999; Heiderstadt et al., 2000; Woodson et al., 2003; Drugan et al., 2005; González- Franco et al., 2017) The concentration of CORT observed after training with the two foot-shock intensities reached levels higher than those reported by Sarabdjitsingh et al. (2009), where intraperitoneal CORT injection of 3 mg/kg led to plasma CORT levels greater than 400 ng/mL, which was enough to induce GR translocation to the nucleus of CA1 cells of the DH, so it was highly likely that GR translocation occurred in the hippocampal cells of the rats trained in the present study. Furthermore, the concentration of free CORT after a stressful task (exposure to a novel environment or forced swimming) takes approximately 20 min to reach its peak in the DH and 90 min in the VH (Droste et al., 2008; Dorey et al., 2012). In this work, we sacrificed animals 60 min after training and we also observed changes in the distribution of the phosphorylated GR variants IR in some of the DH and VH subregions.

Semi-quantification of total GR by Western blotting and by immunohistochemical techniques showed that levels of this protein were not affected 60 min after training in CFC in any of the hippocampal subregions. Several studies have also reported that the optical density (OD) of the total GR protein does not change, either *in vitro* after incubation of U2OS and HEK 293 cell lines with the GR agonist dexamethasone (Wang et al., 2002; Lambert et al., 2013), nor *in vivo* after training animals in fear conditioning (Xing et al., 2014). However, increasing this interval would be likely to induce a decrease in GR concentration in certain subregions of the hippocampus, since GR is a transcription factor that negatively self-regulates in certain brain areas after binding its hormone ligand (Sapolsky et al., 1986).

GR phosphorylation is a post-translational mechanism that affects the location of the receptor within the cell, and therefore regulates its function. Phosphorylation in Ser232 or Ser246 induces the GR to translocate into the nucleus, and regulates (promotes or inhibits) the expression of diverse functional groups of genes, depending on the position of the phosphorylated serine and the time elapsed after activation of the GR. The genes that are regulated by GR can be classified into several functional groups that are involved in cellular metabolism (NADH dehydrogenase subunits 1, 3 and 4, cytochrome b, and lactate dehydrogenase B), regulation of transcription and gene translation mechanisms (chaperone Hsp90, nerve growth factor-induced factor A, and cyclin L1), signal transduction (serotonin 1A receptor, adenosine A1 receptor, and oxytocin receptors), intra- and extracellular trafficking and transport of molecules (synaptosomal-associated protein 25, synaptotagmin I, and choline transporter), neurotransmitter catabolism (monoamine oxidase A), neuron growth (brain-derived neurotrophic factor, nerve growth factor and neurotrophin-3) among other functions (Morsink et al., 2006; Datson et al., 2008; Kadmiel and Cidlowski, 2013). Because phosphorylation is an important step for the transcriptional function of GR, we evaluated changes in pSer232 and pSer246 in the DH and VH.

We obtained contrasting results in the semi-quantification of immunoreactive cells to pSer232 and pSer246 after Western blotting and immunohistochemical analysis, partly because with the first technique we only differentiated between DH and VH, and not among CA1, CA2, CA3, and DG subregions, since they are very small and their extraction would likely lead to contamination of the sample with tissues of neighboring regions. A previous report showed changes in the proportion of both phosphorylated GR variants in nuclear and cytoplasmic fractions of the hippocampus of rats exposed to acute stress (immobility for 30 min; Adzic et al., 2009); however, in this report no differentiation was made between the DH and VH, nor between the CA1, CA2, CA3 or DG subregions. In our case, we investigated possible changes in the phosphorylated variants of GR as related to the functionality of the different hippocampal subregions (as discussed below), so we complemented the results obtained with Western blotting with immunohistochemistry in brain slices, allowing us to quantify their presence and distribution by counting the number of the immunoreactive cells in each hippocampal region using specific antibodies to total GR, pSer232 and pSer246.

We observed that rats trained with the higher foot-shock intensity also showed more hippocampal subregions with increased GR phosphorylation. Several studies report that training with aversive stimuli of high intensities results in memory generalization (a fear response that is triggered by stimuli similar to the CS; Baldi et al., 2004; Dunsmoor et al., 2017; dos Santos Corrêa et al., 2019). In fact, dos Santos Corrêa et al. (2019) speculated that high CORT levels released after training would over-activate the hippocampus to code non-specific contextual features, leading to fear generalization. Our results complement these results by showing that high CORT levels are related to increased GR phosphorylation in the hippocampus, which could lead to the generalization phenomenon.

We observed an increased percentage of immunoreactive cells to pSer246 in the dorsal and ventral regions of the DG and the dorsal region of CA1 after training. The effect of GR phosphorylation on this serine is gene-silencing (Wang et al., 2002; Schoneveld et al., 2004; Galliher-Beckley and Cidlowski, 2009; Hudson et al., 2013). Different molecular techniques to measure changes in gene expression have been shown that one of the first molecular events that occurs in the acquisition of CFC is inhibition of gene expression in the hippocampus, including those genes that are regulated by the GR (angiotensinogen, mineralocorticoid receptor, some ribosomal proteins, lactate dehydrogenase B, and monoamine oxidase A among others), especially within the first 60 min after training or administration of CORT (Morsink et al., 2006; Federighi et al., 2013; Cho et al., 2015). However, these reports did not localize the effect of training on gene-silencing within the different hippocampal subregions. Our work suggests that this effect could be focused mainly on DG.

The DG is the site of entry of information to the hippocampus, especially its ventral region. Its function is to separate out information patterns and to differentiate them before signals are sent to CA3 and then to CA1. Lesions in the dorsal region of the DG impair the acquisition and retrieval of fear memory (Lee and Kesner, 2004), while neurotoxic lesions to the ventral region (specifically in the caudal area) and the subicle diminish the acquisition of CFC (Maren and Holt, 2004). In addition, GR inactivation in the DH or VH with the CORT antagonist RU 38486 just before training impairs fear memory consolidation in the VH (including CA1, CA3, and DG), whereas in the DH it does not affect memory (Donley et al., 2005), suggesting that the effect of GCs on the hippocampus is specific to the activation of GRs in the VH during conditioning. Moreover, DG has been associated with emotional functions because it has direct connections from the amygdala (Blair and Fanselow, 2014). Our results suggest that training in CFC induces GR phosphorylation in Ser246 as a selective mechanism to induce the expression of certain genes to allow cells to transmit specific information (spatial and emotional) of the learning episode to other regions within the hippocampus.

On the other hand, dorsal CA1 is the main information exit site of the hippocampal formation to other areas of the brain, including the retrosplenial and the anterior cingulate cortices, which are mainly involved in the cognitive processing of spatial-visual information and in memory processing (Lee and Kesner, 2004; Fanselow and Dong, 2010). Neurotoxic lesions or optogenetic inhibition in dorsal CA1 block the acquisition and retrieval processes of CFC, indicating that its neurons also encode the context during space exploration (Lee and Kesner, 2004; Maren and Holt, 2004; Ji and Maren, 2008; Goshen et al., 2011; Nomura et al., 2012; Blair and Fanselow, 2014). With these antecedents, it can be proposed that the increase in pSer246 in CA1 could inhibit the expression of genes that are not necessary for the acquisition of fear memory, weakening the transmission of mnemonic information to other brain regions for storage.

In addition, lesion of the basolateral nucleus of the amygdala results in the reduction of nuclear translocation of the GR in the DG and in CA1 (Jeon et al., 2012). Since the functioning of this nucleus is an important component of the neural circuit of fear conditioning, where the sensory information converges and conditioned and unconditioned stimuli are also associated (Phillips and LeDoux, 1992; Goosens and Maren, 2001; Cardinal et al., 2002), the increase in pSer246 that we observed in the DG and CA1 might be modulated by the basolateral amygdala.

We only observed changes in the percentage of immunoreactive cells for pSer232 in CA3 of VH. This area is an association site and a pathway for flowing of information within the hippocampus. Lesions in the ventral CA3 cause deficits in the expression of freezing 24 and 48 h after training (Hunsaker and Kesner, 2008); therefore, this region appears to be important for memory retrieval. It is known that pSer232 induces the expression of GR-regulated genes (corticotropin-releasing hormone receptor 1, nerve growth factor-induced factor A, ribosomal protein S5, and LIM domain kinase 1, among others) by promoting the recruitment of co-activators, as well as the necessary machinery for transcription (Wang et al., 2002; Schoneveld et al., 2004; Datson et al., 2008; Galliher-Beckley and Cidlowski, 2009); however, the increase in the expression of GR-regulated genes occurs 3 h after GR activation (Morsink et al., 2006), so probably the increase in pSer232 that we observed 60 min after training in the CA3 cell nuclei is one of the initial steps towards the expression of genes that code for proteins that will be required for the establishment of long-term memory. In addition, it has been shown that various antidepressants modulate the expression and phosphorylation of GR, altering its translocation to the nucleus and the expression of genes regulated by GR (Anacker et al., 2011; Guidotti et al., 2013). Therefore, we anticipate that the study of phosphorylation, as well as other post-translational modifications that occur in the GR after hormone binding as a result of emotional or stressful learning, will provide key information for understanding the disorders of stress and anxiety, as well as for the generation of new pharmacological strategies.

Our results give rise to new questions: Will the phosphorylation of GR also be observed during the extinction of the fear memory, or does it only occur during its acquisition? Will the acquisition, consolidation, and/or the extinction of fear memory be affected if GR phosphorylation is inhibited on Ser232 and/or Ser246 in those subregions of the hippocampus where we observed changes? Which genes are being modified due to the phosphorylation of GR on these particular serines?

## Conclusion

Our data suggest that one of the mechanisms by which GCs modulate the acquisition of fear memory is through phosphorylation of the GR on Ser232 and Ser246 in specific subregions of the DH and VH, which could regulate the expression of glucocorticoid-responsive genes, which also participate in neuronal plasticity processes.

## Data Availability Statement

The datasets generated for this study are available on request to the corresponding author.

## Ethics Statement

The animal study was reviewed and approved by the Ethics Committee of the Instituto de Neurobiología, Universidad Nacional Autónoma de México.

## Author Contributions

RP-L, ML, and GQ designed research. RP-L, NS, and MC performed research. RP-L, CA, RP-A, ML, and GQ analyzed the data. RP-L, NS, MC, CA, RP-A, ML, and GQ wrote the manuscript.

## Conflict of Interest

The authors declare that the research was conducted in the absence of any commercial or financial relationships that could be construed as a potential conflict of interest.
